# Real-world effectiveness of fostamatinib in patients with immune thrombocytopenia: a single-center retrospective study

**DOI:** 10.1007/s12185-026-04243-w

**Published:** 2026-08-04

**Authors:** Katsumichi Fujimaki, Takaaki Takeda, Kota Kawabata, Ayako Bouno, Yuki Nakajima, Hideaki Nakajima

**Affiliations:** 1 Department of Hematology, Fujisawa City Hospital, 2-6-1, Fujisawa, Fujisawa, Kanagawa 251-8550 Japan; 2 Department of Hematology and Clinical Immunology, Yokohama City University School of Medicine, Kanagawa, Japan

**Keywords:** Combination, Fostamatinib, Immune thrombocytopenia, Spleen tyrosine kinase, Thrombopoietin receptor agonist

## Abstract

**Introduction:**

Immune thrombocytopenia (ITP) is an acquired autoimmune disease characterized by increased platelet destruction and thrombocytopenia. Fostamatinib inhibits spleen tyrosine kinase and suppresses platelet destruction. However, clinical evidence of its efficacy in Japanese patients with ITP remains limited.

**Methods:**

In this study, we retrospectively evaluated the clinical outcomes of fostamatinib, with or without thrombopoietin receptor agonists (TPO-RAs), at a single center in Japan.

**Results:**

The study included 11 patients. At week 1, all 3 evaluable patients (100.0%) achieved platelet count ≥ 30,000/μL and a twofold increase from baseline; at week 2, 10 of 11 (90.9%) met the criteria. Ultimately, 10 of 11 (90.9%) patients achieved the criteria at least once within 24 weeks. All 3 patients using concomitant TPO-RAs (100.0%) achieved the primary endpoint. After fostamatinib initiation, 9 of 11 (81.8%) patients discontinued or reduced their concomitant glucocorticoid (GC) dose. Thrombosis was not observed regardless of concomitant TPO-RA use.

**Conclusions:**

This study demonstrates the real-world effectiveness and safety of fostamatinib in patients with ITP. Platelet count rose soon after fostamatinib initiation, within 1–2 weeks. Fostamatinib may enable reduction or discontinuation of GCs, even in patients with ITP refractory to TPO-RAs.

*Trial registration*: UMIN000057096

**Supplementary Information:**

The online version contains supplementary material available at https://doi.org/10.1007/s12185-026-04243-w.

## Introduction

Immune thrombocytopenia (ITP) is an acquired autoimmune disease in which autoantibodies against platelet membrane glycoproteins bind to platelets and megakaryocytes, resulting in increased platelet destruction and thrombocytopenia [1]. The treatment goal in chronic ITP is to maintain platelet levels ≥30,000/μL, thereby reducing the risk of serious bleeding [2]. In Japan, for patients with ITP who are infected with *Helicobacter pylori* (*H. pylori*), initial therapy is *H. pylori* eradication, which increases platelet counts in 50–70% of patients. Pharmacologic therapy is initiated for patients with ITP without *H. pylori* infection who are at high risk of bleeding or whose platelet counts do not increase during *H. pylori* eradication. These drugs include glucocorticoids (GCs) as first-line therapy, and thrombopoietin receptor agonists (TPO-RAs), rituximab, and splenectomy as second-line options for patients with inadequate response or who are intolerant to GCs [2].

Fostamatinib (FOS) is a spleen tyrosine kinase (Syk) inhibitor, a therapeutic agent for ITP that inhibits the activity of Syk, resulting in the suppression of phagocytosis and destruction of platelets bound to anti-platelet autoantibodies by macrophages [3, 4]. The international consensus report on primary ITP, published in 2019, includes FOS, TPO-RAs, and rituximab as well-evidenced second-line therapies for ITP [5]. However, the “Reference guide for management of adult immune thrombocytopenia in Japan: 2019 Revision” does not include FOS [2] because it had not been approved at the time of publication. In addition, the Spanish ITP Group, published in 2023, recommends TPO-RAs or FOS as second-line treatment and favors FOS in patients with increased thrombosis risk [6].

The efficacy and safety of FOS in patients with ITP have been reported in phase 3 trials (FIT1 and FIT2) [7] and long-term extension studies [8]. A post hoc analysis of phase 3 trials also showed the efficacy of FOS as a second-line therapy for ITP [9]. A 5-year long-term observation study conducted among patients treated with FOS found durable efficacy and safety of FOS, with very low incidence of thromboembolic events [10]. However, clinical evidence in Japanese patients is limited. Only one phase 3 double-blind trial (R788-1301 trial) [11] and its long-term extension studies [12, 13] have been reported. In these clinical trials, concomitant use of TPO-RA, commonly used second-line therapeutic agents for ITP, was not permitted, and early platelet counts within 2 weeks of FOS administration were not measured. Combination therapy with TPO-RAs and FOS [14] and a case series of switching from TPO-RAs to FOS have been reported [15]; however, there is still no evidence regarding combination therapy with TPO-RAs and FOS in Japan, and evidence reflecting real-world post-marketing conditions is lacking.

We aimed to retrospectively investigate the real-world effectiveness and safety of FOS in patients with chronic ITP who received FOS in a single medical institution in Japan (Fujisawa City Hospital) after its launch. We included patients with ITP with or without concomitant TPO-RA use and evaluated the early platelet response within 1–2 weeks after FOS administration. These findings may inform future ITP treatment.

## Materials and methods

### Study design

This single-center, retrospective observational study was conducted at Fujisawa City Hospital in Japan, between March 2025 and April 2025. All study procedures were conducted in accordance with the principles of the Declaration of Helsinki, the Ethical Guidelines for Medical and Biological Research Involving Human Subjects in Japan [16], and other current legal regulations in Japan. The Ethical Review Committee of Fujisawa City Hospital approved the study protocol (approval number: F2024038; approval date: January 21, 2025). This study was registered with the University Hospital Medical Information Network Clinical Trials Registry (UMIN-CTR) (registration number: UMIN000057096) on February 20, 2025, after approval by the ethical review committee, and prior to initiation. We obtained written informed consent from all eligible patients. Data management, monitoring, and statistical analyses were outsourced to a third party (EviPRO Co., Ltd., Tokyo, Japan) to avoid bias and ensure quality.

### Patients

We included (1) patients with chronic ITP; (2) patients who had received FOS at least once in accordance with its package insert after April 2023; (3) patients aged ≥ 18 years; and (4) those who provided written informed consent. We excluded (1) patients who declined access to their medical records and (2) those requiring a legal representative.

### Study intervention and observation

We retrospectively collected information on patients’ characteristics and ITP treatment history (from ITP diagnosis to FOS administration), clinical laboratory values, details of FOS and concomitant ITP therapies, and adverse drug reactions (ADRs) recorded from FOS initiation to the date of study approval by the ethical review committee. For patients with multiple visits during the study period, all medical information was collected.

### Outcomes

The pre-specified endpoints in this study were as follows: (1) time course of platelet count in each patient, (2) proportion of patients who achieved a platelet count ≥ 30,000 /μL and a twofold increase from baseline at least once within 1, 2, 4, 8, 12, 16, 20, and 24 weeks after initiation of FOS, (3) proportion of patients whose platelet count was ≥ 30,000/μL and showed a twofold increase from baseline at each observation point, (4) proportion of patients whose platelet count was ≥ 50,000/μL at each observation point, (5) proportion of patients whose platelet count was ≥ 100,000/μL at each observation point, (6) duration of platelet response in each patient, (7) time required to achieve and maintain a platelet count ≥ 50,000/μL, and (8) frequency of bleeding events from FOS initiation to week 24. The safety endpoints were the frequency of ADRs. We performed post hoc exploratory analyses of hematological indices (mean platelet volume [MPV] and platelet distribution width [PDW]) for correlation with changes in platelet count. Note that the endpoints (2) and (3) were based on the definition of “Response” in the International Working Group Criteria (platelet count ≥ 30 × 10^9^/L and at least twofold increase the baseline count and absence of bleeding) [17]. This study employed these platelet criteria except for the bleeding criterion.

### Statistical analysis

All tests were two-sided, and a *p*-value < 0.05 was considered statistically significant. Missing values were not imputed. A statistical analysis plan was developed prior to the database lock. All statistical analyses were outsourced to a third-party (EviPRO Co., Ltd.) to avoid bias and ensure quality. All statistical analyses were performed using SAS version 9.4 (SAS Institute Inc., Cary, NC, USA).

All enrolled patients were included in the analysis; however, we excluded patients who were ineligible after enrollment or who had no data after FOS initiation.

Patient characteristics were reported as frequencies and proportions for categorical data and as summary statistics (number of patients, minimum, first quartile, median, third quartile, and maximum) for continuous data. For the endpoint 1, we plotted the time course of the platelet count in each patient on a line graph, with time course of dose of FOS and concomitant ITP treatment agents. We also calculated the summary statistics at each observation point in the analysis set, and plotted the median of the platelet count in each observation point on a line graph, with stratified analyses of patients with or without concomitant TPO-RAs use at FOS initiation and treatment line (sequence of FOS administration among ITP therapeutic agents). For the endpoint 2, we calculated the number and proportion of patients who attained a platelet count ≥ 30,000/μL and a twofold increase from baseline at least once within 1, 2, 4, 8, 12, 16, 20, and 24 weeks after FOS initiation. For the endpoints 3–5, we calculated the number and proportion of patients and conducted stratified analyses of patients with or without concomitant TPO-RAs use at FOS initiation and treatment line. For the endpoint 6, we plotted the duration of platelet response in each patient on a swimmer plot. For the endpoint 7, we calculated the number of days required to achieve and maintain a platelet count ≥ 50,000/μL and conducted summary statistics for the analysis set. For secondary endpoint 8, we calculated the number and frequency of patients with bleeding events. For the safety endpoint, we calculated the number and frequency of patients with each ADR to FOS. Severity of ADR was classified based on physician assessment. For the exploratory analysis of MPV and PDW, we used summary statistics (number of patients, minimum, first quartile, median, third quartile, and maximum). Additionally, we used Spearman’s rank correlation coefficient to assess the correlation between the change in platelet count and that in MPV or PDW from baseline.

## Results

### Baseline participant characteristics

From the launch of FOS in Japan (April 2023) until the data cut-off date when this study protocol was approved by the ethical review board (21 January 2025), 12 patients received FOS at Fujisawa City Hospital in routine clinical practice, and provided written informed consent for this study. One patient was excluded after peer review because chronic ITP could not be confirmed at the initiation of FOS therapy (inclusion criterion (1)), and finally 11 patients were included in our analysis. Notably, no patients discontinued FOS treatment during the study period.

The baseline patient characteristics in all patients and each patient are presented in Table 1 and supplementary Table 1, respectively. Among the 11 patients, 8 (72.7%) were female. The median age was 73.0 years (range: 35–80 years), and the median platelet count at FOS initiation was 22,000/μL (range: 3000–74,000/μL). Nine patients (81.8%) had at least one risk factor for thrombosis, including one patient (9.1%) positive for antiphospholipid antibodies. TPO-RAs were administered to six patients (54.5%) before FOS initiation. Subsequently, three patients (27.3%) received TPO-RAs in combination with FOS. No patient had history of splenectomy. Because three patients were *H. pylori* positive, *H. pylori* eradication had been conducted before the FOS administration. All 11 patients met the criteria of “GC responsiveness”, defined as the maximum effect within one month after initiation of GC was a platelet count ≥ 30,000/μL and a twofold or more increase from the previous value. FOS was used as second-, third-, or fourth-line therapy for ITP in three (27.3%), seven (63.6%), and one (9.1%) patients, respectively. The median duration of FOS administration was 7.5 months (range: 3.0–19.5 months).

**Table 1:** Demographic and clinical characteristics of patients at baseline (n = 11)

Variables	Frequency (%)
Sex (male/female)	3 (27.3)/8 (72.7)
Age at first administration of FOS (years)	73.0 [35, 80]
Platelet (/μL)	22,000 [3000, 74,000]
< 30,000	6 (54.5)
30,000–50,000	1 (9.1)
≥ 50,000	4 (36.4)
Risk factor for thrombosis	9 (81.8)
Hypertension	5 (45.5)
Diabetes mellitus	3 (27.3)
Dyslipidemia	2 (18.2)
Positive antiphospholipid antibody	1 (9.1)
Smoking	1 (9.1)
History of thrombosis	0 (0.0)
Obesity	0 (0.0)
*Prior treatment medication for ITP*	
Glucocorticoid	11 (100.0)
TPO-RA	6 (54.5)
Rituximab	1 (9.1)
IVIg	2 (18.2)
Other	0 (0.0)
History of splenectomy	0 (0.0)
*Treatment lines with FOS*	
Second line	3 (27.3)
Third line	7 (63.6)
Fourth line	1 (9.1)
Concomitant treatment with TPO-RAs	3 (27.3)
*Reason for FOS administration*	
Platelet count < 30,000/μL	6 (54.5)
Dose reduction or withdrawal of other medications for ITP	4 (36.4)
Other*	1 (9.1)

Data are presented as the median (range [minimum, maximum]) for continuous variables and as the number of patients (%) for categorical variables

^*^To maintain a high platelet count due to repeated retinal hemorrhage

FOS, fostamatinib; ITP, immune thrombocytopenia; TPO-RA, thrombopoietin receptor agonist; IVIg, intravenous immunoglobulin

### Dose of FOS

All 11 patients received an initial FOS dose of 200 mg/day. During the observation period, the FOS dose was adjusted or FOS was discontinued by the attending physician based on the patient’s platelet count and/or blood pressure.

### Effectiveness

**Fig. 1:**
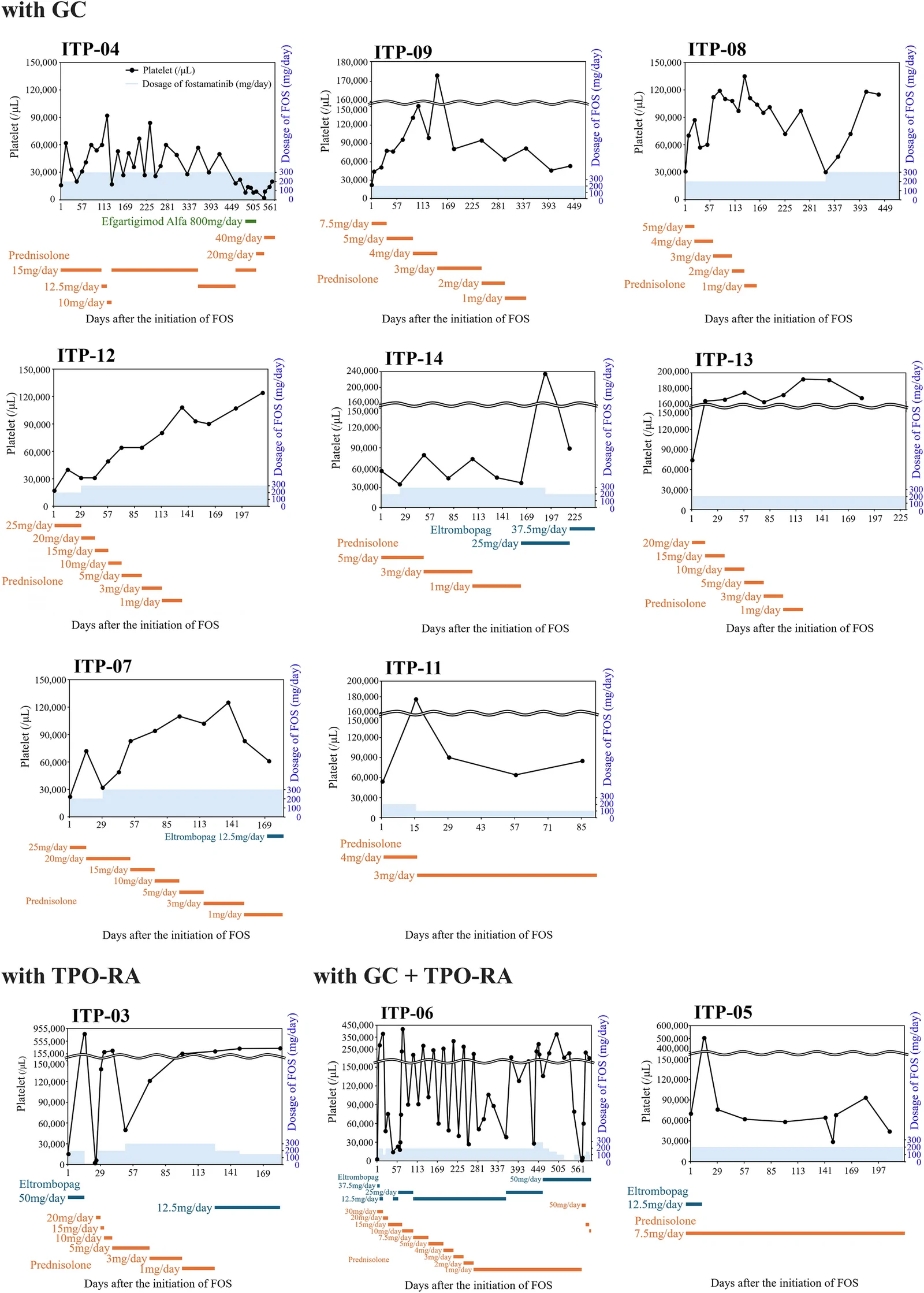
Time course of treatment and platelet count in each patient. The time course of the platelet count during FOS treatment is presented as a line graph, and the dosage of FOS during the treatment course is shown in the area graph. Dosages and durations of other concomitant medications for ITP are shown as bars below the graph. FOS: fostamatinib, GC: glucocorticoid, TPO-RA: thrombopoietin receptor agonist

Time courses of treatments and the platelet count in each patient are shown in Fig. 1. In most patients (10 of 11 patients (90.9%), except ITP-14), platelet count increased within the 1–2 weeks after initiation of FOS. Among the three patients who received FOS as second-line therapy for ITP (ITP-04, ITP-12, and ITP-13), all were treated using the combination therapy with GCs; two patients (ITP-12 and ITP-13) discontinued or reduced the GCs within 24 weeks after FOS initiation. Among the eight patients who received FOS as third-line therapy or later (ITP-03, ITP-05, ITP-06, ITP-07, ITP-08, ITP-09, ITP-11, and ITP-14), seven (ITP-03, ITP-06, ITP-07, ITP-08, ITP-09, ITP-11, and ITP-14) discontinued or reduced concomitant GC use. Overall, 9/11 patients (81.8%) discontinued or reduced concomitant GC use within 24 weeks of FOS initiation.

**Fig. 2:**
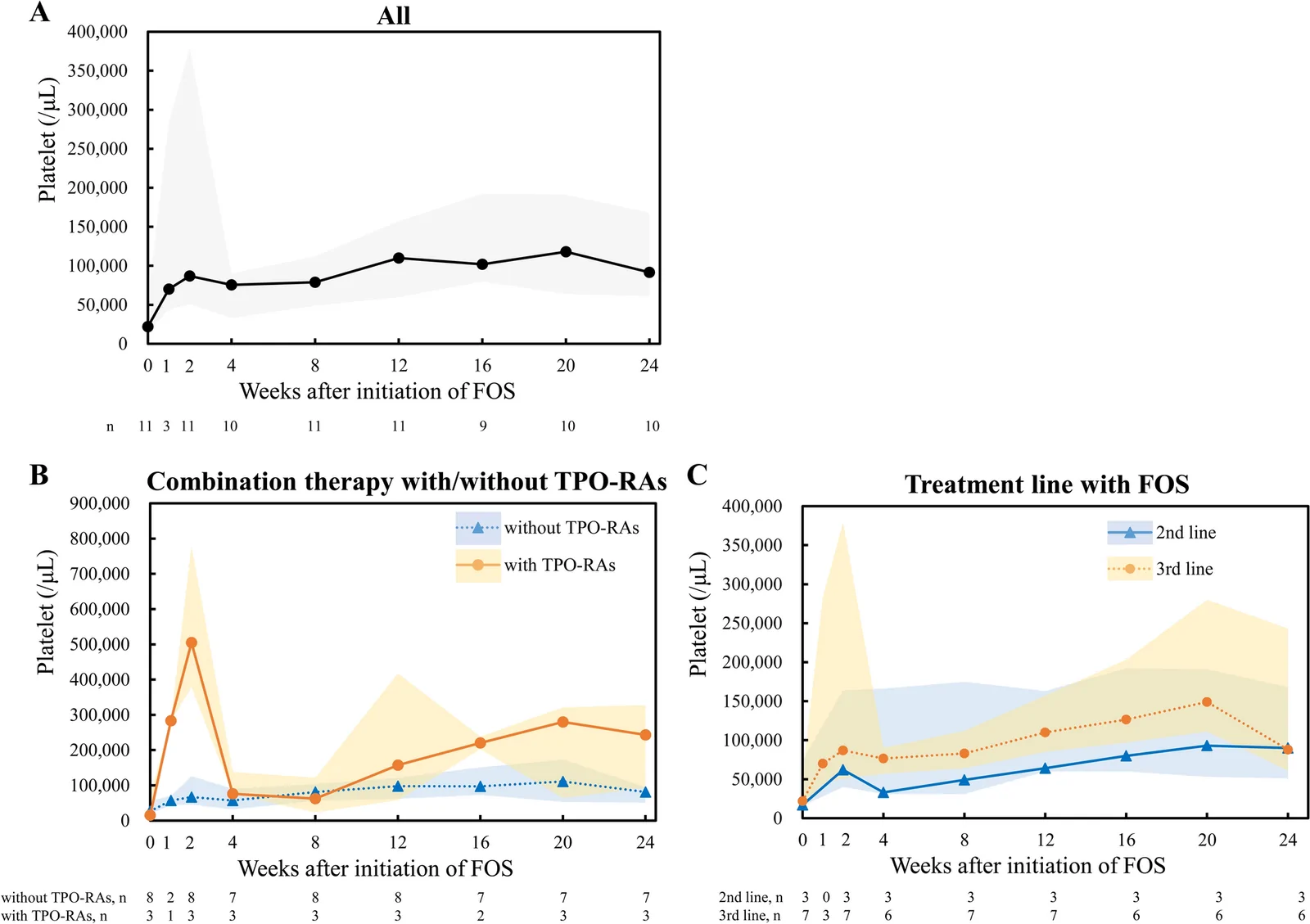
Change in median platelet count after FOS initiation. A: All registered patients; B: Subgroup analysis with/without combination therapy with TPO-RAs; C: Subgroup analysis based on the treatment line with FOS. Data are presented as median as a line graph and interquartile range as an area graph. FOS: fostamatinib, TPO-RA: thrombopoietin receptor agonist

As platelet counts after FOS initiation varied greatly among individuals, Fig. 2 and Supplementary Fig. 1 present the changes in the median and interquartile range of the platelet count as a line graph and an area graph, respectively. In all patients, platelet levels rapidly increased after FOS initiation, exceeding 50,000/μL, and the platelet level was maintained or gradually increased until week 24 (Fig. 2A). The platelet levels increased and were maintained in the subgroup without combination therapy with TPO-RAs, whereas they tentatively showed high platelet levels at week 2 in the subgroup with combination therapy with TPO-RAs (Fig. 2B). When stratified by FOS treatment lines, the tendency for rapid increase and maintenance of platelet levels after FOS initiation was similar among the treatment lines (Fig. 2C). A similar tendency was observed in patients whose platelet count was < 30,000/μL at baseline (Supplementary Fig. 1).

**Fig. 3:**
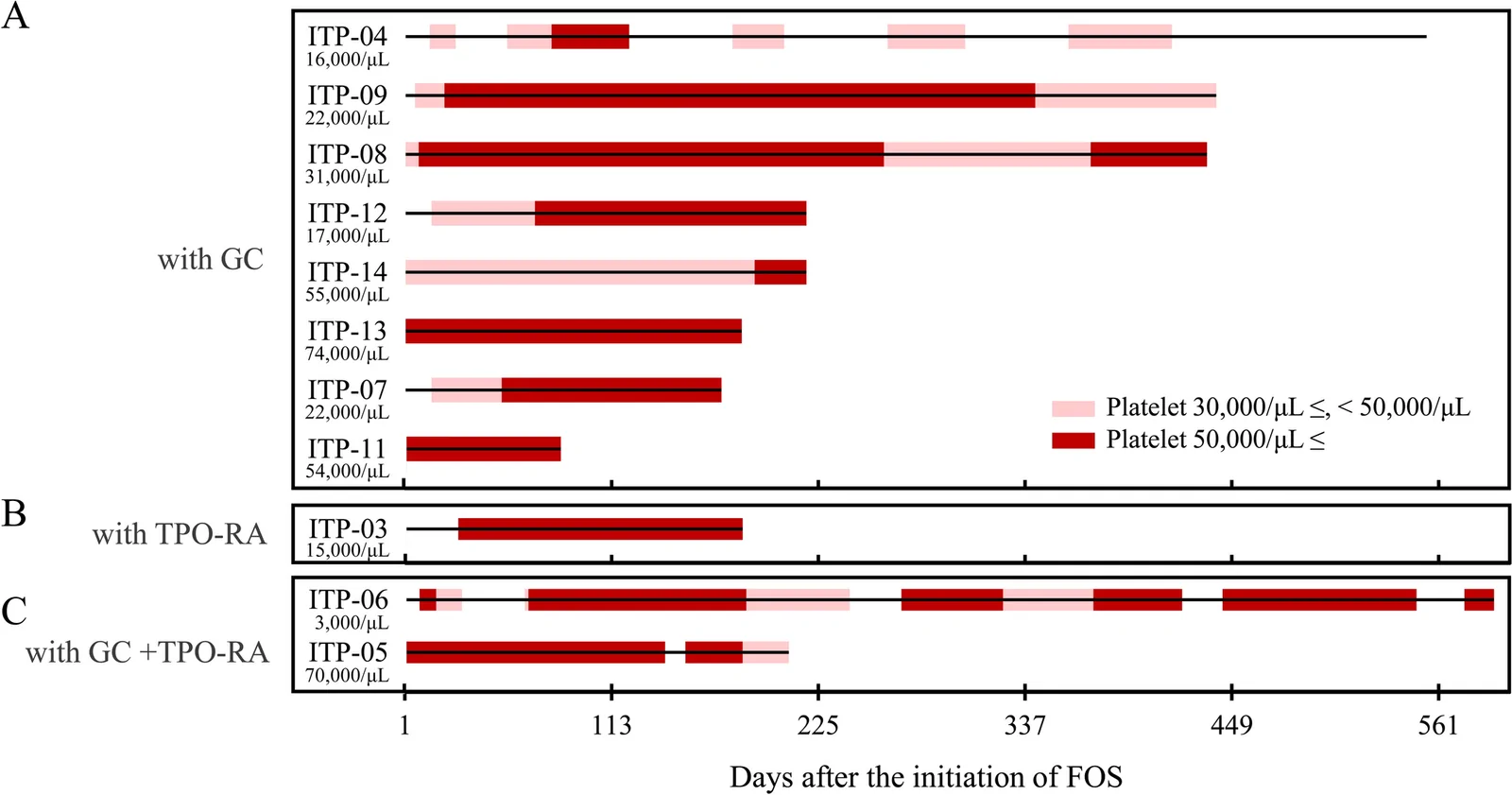
Swimmer plots show the individual duration of platelet response after the initiation of FOS. A: GC, B: TPO-RA, C: GC + TPO-RA. FOS: fostamatinib, GC: glucocorticoids, TPO-RA: thrombopoietin receptor agonist. Platelet count at the start of FOS administration is shown as the number of cases

Swimmer plots of individual duration of platelet response after FOS initiation are shown in Fig. 3. Most patients maintained platelet counts ≥ 30,000/μL after FOS initiation, and many maintained ≥ 50,000/μL. Among four patients who received FOS for ≥ 12 months (ITP-04, ITP-09, ITP-08, and ITP-06), two (ITP-08 and ITP-06) maintained platelet counts ≥ 50,000/μL after 12 months, and one (ITP-09) maintained platelet counts ≥ 30,000 /μL and a twofold increase from baseline.

Among the 11 patients, 10 (90.9%) achieved a platelet count ≥ 30,000/μL and twofold increase from baseline at least once within 24 weeks after initiation of FOS (Supplementary Fig. 2A). One patient showed an increase in platelet count after dose of FOS (from 55,000/μL at baseline to 79,000/μL at week 8), however, did not achieve the criteria due to decreased platelet count by reducing the dose of concomitant GC. Three of 3 (100.0%) and 10 of 11 (90.9%) patients achieved a platelet count ≥ 30,000 /μL and a twofold increase from baseline at weeks 1 and 2, respectively (Supplementary Fig. 3A). With concomitant TPO-RAs use, all 3 patients (100.0%) achieved the criteria (Supplementary Table 2). Regardless of the combination therapy with TPO-RAs or FOS as second- or third-line therapy for ITP, the proportion of patients who met platelet endpoints were high, especially at week 2 and after week 12.

Four patients with platelet counts ≥ 50,000/μL at baseline were excluded, and we calculated the proportion of patients who had platelet counts ≥ 50,000/μL after FOS initiation. After week 12, all seven patients (100%) experienced a platelet count ≥ 50,000/μL (Supplementary Fig. 2B, Supplementary Fig. 3B). In the subgroup analysis based on treatment options, the proportion of patients achieving a platelet count ≥ 50,000/μL was 100% at most observation points regardless of the use of TPO-RAs combination therapy or FOS use as second- or third-line therapy (Supplementary Table 2).

At week 2, five patients (5/11 patients, 45.5%) achieved a platelet count ≥ 100,000/μL after FOS initiation (Supplementary Fig. 3C), and eight (8/11 patients, 72.7%) achieved it at least once after week 12 (Supplementary Fig. 2C). In the subgroup analysis, the proportion of patients achieving a platelet count ≥ 100,000/μL was ≥ 50% at several observation points regardless of the use of combination therapy with TPO-RAs or treatment lines for ITP, or FOS use as second- or third-line therapy (Supplementary Table 2).

The median duration to achieve a platelet count ≥ 50,000/μL was 15.0 days (range: 8.0–71.0 days), and to maintain it during the observation period was 155.0 days (range: 43.0–357.0 days), respectively.

In total, four patients (36.4%) experienced bleeding events (Table 2): purpura in three (27.3%) and oral hemorrhage in one (9.1%). All bleeding events were mild, and the patients recovered.

**Table 2:** Bleeding events

	Number of patients (%)
Number of patients analyzed	11
Bleeding event	4 (36.4)
Purpura	3 (27.3)
Oral hemorrhage	1 (9.1)

Data are presented as the number of patients (%)

### Safety

FOS-related ADRs occurred in 9 patients (81.8%) (Table 3). The most frequent ADRs were hypertension in six patients (54.5%), diarrhea in four patients (36.4%), and hepatic dysfunction in four patients (36.4%). Hypertension was diagnosed by the attending physician when antihypertensive agents were added or uptitrated in patients presenting a complication of hypertension. Hypertension was mild in five patients and severe in one patient that the measured blood pressure values corresponded to grade 3 according to the Common Terminology Criteria for Adverse Events (CTCAE). As a measure for these FOS-related ADRs, the dose of FOS was temporary reduced in four patients (ITP-03 and ITP-14 due to diarrhea and ITP-06 and ITP-11 due to hypertension) and temporary discontinued in one patient (ITP-06 due to hypertension). No thrombotic events were observed regardless of combination therapy with TPO-RAs.

**Table 3:** Adverse drug reactions

	Number of patients (%)			
Number of patients analyzed	11			
	Total	Mild	Moderate	Severe
Hypertension	6 (54.5)	5 (45.5)	0 (0.0)	1 (9.1)
Diarrhea	4 (36.4)	4 (36.4)	0 (0.0)	0 (0.0)
Hepatic dysfunction	4 (36.4)	4 (36.4)	0 (0.0)	0 (0.0)
Increase in serum creatinine	1 (9.1)	1 (9.1)	0 (0.0)	0 (0.0)
Headache	1 (9.1)	0 (0.0)	0 (0.0)	1 (9.1)

Data are presented as the number of patients (%)

Severity was classified based on physician assessment

### Hematological tests

After FOS initiation, the median platelet count increased from 22,000/μL at baseline to 91,500/μL at week 24. Among the eight patients with MPV and PDW data available before starting FOS, there was a reduction in MPV from 11.6 fL at baseline to 11.2 fL at week 24, and in PDW from 14.9 to 13.5 fL. On correlation analysis, we found a negative correlation between changes in platelet count and changes in MPV or PDW throughout the observation period (Supplementary Table 3). Furthermore, there were significant correlations in MPV at weeks 8 and 12 after FOS initiation and in PDW at weeks 8, 12, and 24.

## Discussion

In this retrospective observational study, 10/11 patients (90.9%) had reached a platelet count ≥ 30,000/μL and a twofold increase from baseline at week 2 of FOS administration. This corresponds to the platelet criteria of partial response according to the “Reference guide for management of adult immune thrombocytopenia in Japan: 2019 Revision” [2]. These findings are consistent with or even better than those of a previous study on combination therapy with TPO-RA (avatrombopag) and FOS (60% at week 2 and 83.3% overall) [14]. Moreover, 3/3 patients (100.0%) reached a platelet count ≥ 30,000/μL and a twofold increase from baseline at week 1. The median time for an early platelet response after FOS administration is reported to be 9 days [18]. Our results are consistent with this, as we observed an increase in platelet counts within 1–2 weeks after FOS initiation. Therefore, FOS can be considered a therapeutic agent for ITP that obtains an early platelet response. However, given the variations in patient backgrounds, selection criteria, and study designs among the data used for comparison, it is essential to approach the interpretation of this study’s efficacy with caution.

Among patients receiving combination therapy with FOS and GCs, 9 (81.8%) discontinued or reduced the dose of GCs within 24 weeks of FOS administration. Although GCs are effective therapeutic agents for ITP, their multiple side effects outweigh their benefits in the long term [5], promoting dose reductions or early discontinuation in routine practice in Japan, especially with newer treatment options such as TPO-RAs [19]. Combined with a previous report that long-term administration of FOS reduced the dose of or led to the discontinuation of GCs [13], FOS could be a treatment option to reduce GC-associated side effects. Based on our treatment experience at Fujisawa City Hospital, cases that respond to GCs are highly likely to be ITP, while cases that do not respond to GCs could be diseases other than ITP, such as aplastic anemia or myelodysplastic syndrome (MDS). Response to GCs was included as an eligibility criterion in a trial of rilzabrutinib in patients with ITP [20]. The early efficacy of FOS observed in this study may be partly attributable to the inclusion of patients with ITP who were responsive to GCs. The combination of FOS with GCs could potentially result in a greater increase in platelet count in GC-responsive patients.

All three patients on combination therapy with FOS and TPO-RA reached a platelet count ≥ 30,000 /μL with a twofold increase from baseline at week 2, and two of them (66.7%) at week 24. These results are consistent with previous reports on combination therapy with FOS and TPO-RAs [14, 15]. Owing to the difference in the mode of action, combination therapy with FOS and TPO-RAs is expected to be effective in increasing platelet counts, even in refractory cases. However, as shown in Fig. 2B, the addition of FOS in patients who have been treated with TPO-RAs resulted in a tentatively high platelet level in the early phase of FOS administration (at week 1–2), which might increase the risk of thrombosis. Therefore, it is important to monitor platelet counts during the early stages of FOS administration in patients concomitantly on TPO-RA.

Consistent with a previous report on the efficacy of FOS as second-line therapy, all three patients on FOS had an increase in platelet counts [9]. Among eight patients receiving FOS as third-line or later therapy, seven (87.5%) achieved the primary endpoint and showed a high proportion of achievement after week 2. Although a previous report showed better efficacy of FOS as second-line than as third-line therapy [9], we found comparable efficacy of FOS even in refractory cases.

The median duration to achieve platelet count ≥ 50,000/μL in patients on FOS was 8.0 days, which was similar to that in patients on efgartigimod [21] and avatrombopag [22]. Regarding the long-term efficacy of FOS, among four patients who received FOS for ≥ 12 months, two patients maintained platelet counts ≥ 50,000/μL after 12 months, and one patient maintained platelet counts ≥ 30,000/μL and a twofold increase from baseline. One patient failed to maintain platelet count and was GC-dependent. This patient tested positive for antiphospholipid antibodies and was "triple positive," with all of anticardiolipin, anti-β2-glycoprotein I, and lupus anticoagulant being positive, although this patient did not meet the diagnostic criteria of anti-phospholipid antibody syndrome. It has been reported that patients with triple positive antiphospholipid antibodies tend to have lower platelet count and may face more difficulties in treatment than non-triple positive patients [23]. Therefore, confirming the antiphospholipid antibody profile in patients with ITP is considered beneficial for understanding the pathology and treatment.

MPV and PDW have been reported to be high in patients with ITP [24, 25] due to an increase in the proportion of immature large platelets owing to the enhancement of platelet destruction. We found lower MPV and PDW levels after FOS initiation. This reduction in MPV and PDW levels may reflect the suppression of platelet destruction by FOS. These results suggest that MPV and PDW levels can be used as indicators to help select second-line therapeutic agents after first-line GC treatment. Given the exploratory nature of these results, it is essential to conduct additional research with more cases.

Hypertension (54.5%), diarrhea (36.4%), and hepatic dysfunction (36.4%) were observed as an ADR in this study. The proportion of hypertension was higher than that reported in a phase 3 trial in Japan (27.3%) [11]. Possible reasons for the higher frequency of hypertension might include older age (median, 73.0 years; range: 35–80 years in this study vs median, 61 years; range: 25–81 years in the phase 3 trial in Japan) and the high proportion of patients with hypertension as a complication at baseline (5/11 patients in this study vs. 8/22 patients in the phase 3 trial in Japan—data not shown). Some of ADRs observed in this study could be controlled by temporary withdrawal of FOS or the addition of therapeutic agents, demonstrating that FOS could be continued while managing the ADRs.

This study has several limitations. First, it was conducted at a single medical institution, which limits generalizability. Second, because this was a retrospective study, data were not available at all observation points for all patients. Third, this study was conducted in Japan, and most registered patients, except one, were Japanese. This might limit the generalizability of the results of this study. Fourth, the sample size was small (11 patients); this is because ITP is a rare disease, and this study was conducted in a single medical institution. Fifth, given the establishment of comparatively permissive reaction criteria, the efficacy results should be interpreted with caution when compared to existing reports.

In conclusion, this retrospective study demonstrated the real-world efficacy and safety of FOS in increasing and maintaining platelet levels in patients with chronic ITP in Japan. In particular, this study revealed an early platelet response–1–2 weeks after FOS initiation. FOS might be a therapeutic option when discontinuing or reducing GCs, and its combination with a TPO-RA, which has a different mechanism of action, may be effective even in refractory cases.

## Supplementary Information

Below is the link to the electronic supplementary material.

Supplementary file1 (DOCX 377 KB)

## Data Availability

The datasets generated and/or analyzed during the current study are not publicly available because of the lack of a statement enabling data sharing with a third party after the end of the study in the study protocol, informed consent documents, and lack of approval for data sharing by the ethics review board. Anonymized datasets may be shared with interested researchers upon request. Prior to data provision, the following steps may be required: development of a new protocol, approval from an appropriate ethical review board, legally required informed consent procedures, and data provision contracts or material transfer agreements, if required.
