# Supplementary material for: Real-world effectiveness of fostamatinib in patients with immune thrombocytopenia: a single-center retrospective study
Source: Int J Hematol. 2026 Aug 4;124(5):630–9. doi: 10.1007/s12185-026-04243-w (PMC13719711; doi:10.1007/s12185-026-04243-w)
Supplement: Supplementary file 1 [file 12185_2026_4243_MOESM1_ESM.docx]

**Supplementary information**

**Title:**

Real-world effectiveness of fostamatinib in patients with immune thrombocytopenia: A single-center retrospective study

**Authors:**

Katsumichi Fujimaki^1,*^, Takaaki Takeda^1^, Kota Kawabata^1^, Ayako Bouno^1^, Yuki Nakajima^1^, Hideaki Nakajima^2^

**Affiliations:**

^1^ Department of Hematology, Fujisawa City Hospital, Kanagawa, Japan

^2^ Department of Hematology and Clinical Immunology, Yokohama City University School of Medicine, Kanagawa, Japan

***Corresponding author:**

Katsumichi Fujimaki

Department of Hematology, Fujisawa City Hospital, 2‑6‑1, Fujisawa, Fujisawa, Kanagawa 251‑8550, Japan

Supplementary Table 1. Demographic and clinical characteristics of each patient at baseline

| ID | ITP-03 | ITP-04 | ITP-05 | ITP-06 | ITP-07 | ITP-08 | ITP-09 | ITP-11 | ITP-12 | ITP-13 | ITP-14 |
| --- | --- | --- | --- | --- | --- | --- | --- | --- | --- | --- | --- |
| Age | 76 | 47 | 54 | 48 | 77 | 75 | 35 | 72 | 73 | 74 | 80 |
| Sex | female | female | male | female | male | female | female | female | male | female | female |
| Duration of ITP (years) | 3.4 | 16.4 | 11.1 | 16.4 | 4.6 | 11.7 | 2.7 | 33.8 | 0.9 | 0.3 | 19.3 |
| Platelet (/μL) | 15,000 | 16,000 | 70,000 | 3,000 | 22,000 | 31,000 | 22,000 | 54,000 | 17,000 | 74,000 | 55,000 |
| Risk factors for thrombosis |  |  |  |  |  |  |  |  |  |  |  |
| Hypertension | － | － | － | ◯ | ◯ | － | ◯ | ◯ | － | ◯ | － |
| Diabetes mellitus | ◯ | － | － | － | － | － | － | － | ◯ | － | ◯ |
| Dyslipidemia | － | － | － | － | － | － | － | － | － | ◯ | ◯ |
| Positive aPL | － | ◯ | － | － | － | － | － | － | － | － | － |
| Smoking | － | － | － | － | － | － | － | － | ◯ | － | － |
| History of thrombosis | － | － | － | － | － | － | － | － | － | － | － |
| Obesity | － | － | － | － | － | － | － | － | － | － | － |
| History of splenectomy | － | － | － | － | － | － | － | － | － | － | － |
| Types of medication for ITP from diagnosis to 4 weeks before initiation of FOS |  |  |  |  |  |  |  |  |  |  |  |
| Glucocorticoids | － | ◯ | ◯ | ◯ | ◯ | ◯ | ◯ | ◯ | ◯ | ◯ | ◯ |
| TPO-RAs | ◯ | － | ◯ | ◯ | － | ◯ | ◯ | ◯ | － | － | － |
| Rituximab | － | － | ◯ | － | － | － | － | － | － | － | － |
| IVIg | － | － | － | － | ◯ | － | － | － | － | － | ◯ |
| Others | － | － | － | － | － | － | － | － | － | － | － |
| Dose of medication for ITP within 4 weeks before initiation of FOS |  |  |  |  |  |  |  |  |  |  |  |
| Prednisolone (mg/day) |  | 15 | 7.5 | 40 | 25 | 5 | 7.5 | 4 | 25 | 20 | 5 |
| Eltrombopag (mg/day) | 50 |  | 12.5 | 37.5 |  |  |  |  |  |  |  |
| Concomitant medication for ITP at initiation of FOS |  |  |  |  |  |  |  |  |  |  |  |
| Prednisolone (mg/day) |  | 15 | 7.5 | 30 | 25 | 5 | 7.5 | 4 | 25 | 20 | 5 |
| Eltrombopag (mg/day) | 50 |  | 12.5 | 37.5 |  |  |  |  |  |  |  |
| Initial dose of FOS (mg/day) | 200 | 200 | 200 | 200 | 200 | 200 | 200 | 200 | 200 | 200 | 200 |
| Treatment lines with FOS | Third | Second | Fourth | Third | Third | Third | Third | Third | Second | Second | Third |
| Reason for FOS administration |  |  |  |  |  |  |  |  |  |  |  |
| Platelet count <30,000~~(~~/μL | ◯ | ◯ |  | ◯ | ◯ |  | ◯ |  | ◯ |  |  |
| Dose reduction or withdrawal of other medications for ITP |  |  | ◯ |  |  | ◯ |  |  |  | ◯ | ◯ |
| Other* |  |  |  |  |  |  |  | ◯ |  |  |  |

* To maintain a high platelet count due to repeated retinal hemorrhage.

FOS, fostamatinib; ITP, immune thrombocytopenia; TPO-RA, thrombopoietin receptor agonist; aPL, antiphospholipid antibody; IVIg, intravenous immunoglobulin

Supplementary Table 2. Subgroup analysis of proportion of patients who met platelet endpoints

| Population | Observation point | Platelet count ≥30,000 /μL and twofold increase from baseline | | Platelet count ≥50,000 /μL | | Platelet count ≥100,000 /μL | |
| --- | --- | --- | --- | --- | --- | --- | --- |
|  |  | N | n (%) | N | n (%) | N | n (%) |
| With TPO-RAs |  |  |  |  |  |  |  |
|  | Week 1 | 1 / 3 | 1 (100.0) | 1 / 2 | 1 (100.0) | 1 / 3 | 1 (100.0) |
|  | Week 2 | 3 / 3 | 3 (100.0) | 2 / 2 | 2 (100.0) | 3 / 3 | 3 (100.0) |
|  | Week 4 | 3 / 3 | 2 (66.7) | 2 / 2 | 2 (100.0) | 3 / 3 | 1 (33.3) |
|  | Week 8 | 3 / 3 | 1 (33.3) | 2 / 2 | 1 (50.0) | 3 / 3 | 1 (33.3) |
|  | Week 12 | 3 / 3 | 2 (66.7) | 2 / 2 | 2 (100.0) | 3 / 3 | 2 (66.7) |
|  | Week 16 | 2 / 3 | 2 (100.0) | 2 / 2 | 2 (100.0) | 2 / 3 | 2 (100.0) |
|  | Week 20 | 3 / 3 | 2 (66.7) | 2 / 2 | 2 (100.0) | 3 / 3 | 2 (66.7) |
|  | Week 24 | 3 / 3 | 2 (66.7) | 2 / 2 | 2 (100.0) | 3 / 3 | 2 (66.7) |
| Without TPO-RAs |  |  |  |  |  |  |  |
|  | Week 1 | 2 / 8 | 2 (100.0) | 2 / 5 | 1 (50.0) | 2 / 8 | 0 (0.0) |
|  | Week 2 | 8 / 8 | 7 (87.5) | 5 / 5 | 4 (80.0) | 8 / 8 | 2 (25.0) |
|  | Week 4 | 7 / 8 | 3 (42.9) | 5 / 5 | 2 (40.0) | 7 / 8 | 1 (14.3) |
|  | Week 8 | 8 / 8 | 5 (62.5) | 5 / 5 | 3 (60.0) | 8 / 8 | 2 (25.0) |
|  | Week 12 | 8 / 8 | 6 (75.0) | 5 / 5 | 5 (100.0) | 8 / 8 | 4 (50.0) |
|  | Week 16 | 7 / 8 | 6 (85.7) | 5 / 5 | 5 (100.0) | 7 / 8 | 3 (42.9) |
|  | Week 20 | 7 / 8 | 6 (85.7) | 5 / 5 | 5 (100.0) | 7 / 8 | 4 (57.1) |
|  | Week 24 | 7 / 8 | 6 (85.7) | 5 / 5 | 5 (100.0) | 7 / 8 | 1 (14.3) |
| FOS as second line therapy |  |  |  |  |  |  |  |
|  | Week 1 | 0 / 3 | 0 (-) | 0 / 2 | 0 (-) | 0 / 3 | 0 (-) |
|  | Week 2 | 3 / 3 | 3 (100.0) | 2 / 2 | 1 (50.0) | 3 / 3 | 1 (33.3) |
|  | Week 4 | 3 / 3 | 2 (66.7) | 2 / 2 | 0 (0.0) | 3 / 3 | 1 (33.3) |
|  | Week 8 | 3 / 3 | 2 (66.7) | 2 / 2 | 0 (0.0) | 3 / 3 | 1 (33.3) |
|  | Week 12 | 3 / 3 | 3 (100.0) | 2 / 2 | 2 (100.0) | 3 / 3 | 1 (33.3) |
|  | Week 16 | 3 / 3 | 3 (100.0) | 2 / 2 | 2 (100.0) | 3 / 3 | 1 (33.3) |
|  | Week 20 | 3 / 3 | 3 (100.0) | 2 / 2 | 2 (100.0) | 3 / 3 | 1 (33.3) |
|  | Week 24 | 3 / 3 | 3 (100.0) | 2 / 2 | 2 (100.0) | 3 / 3 | 1 (33.3) |
| FOS as third line therapy |  |  |  |  |  |  |  |
|  | Week 1 | 3 / 7 | 3 (100.0) | 3 / 5 | 2 (66.7) | 3 / 7 | 1 (33.3) |
|  | Week 2 | 7 / 7 | 6 (85.7) | 5 / 5 | 5 (100.0) | 7 / 7 | 3 (42.9) |
|  | Week 4 | 6 / 7 | 3 (50.0) | 5 / 5 | 4 (80.0) | 6 / 7 | 1 (16.7) |
|  | Week 8 | 7 / 7 | 4 (57.1) | 5 / 5 | 4 (80.0) | 7 / 7 | 2 (28.6) |
|  | Week 12 | 7 / 7 | 5 (71.4) | 5 / 5 | 5 (100.0) | 7 / 7 | 5 (71.4) |
|  | Week 16 | 6 / 7 | 5 (83.3) | 5 / 5 | 5 (100.0) | 6 / 7 | 4 (66.7) |
|  | Week 20 | 6 / 7 | 5 (83.3) | 5 / 5 | 5 (100.0) | 6 / 7 | 5 (83.3) |
|  | Week 24 | 6 / 7 | 5 (83.3) | 5 / 5 | 5 (100.0) | 6 / 7 | 2 (33.3) |

N represents the number of patients with measured values / number of patients in the population.

Data are presented as the number of patients (%).

FOS: fostamatinib, TPO-RA: thrombopoietin receptor agonist.

Supplementary Table 3. Correlation between changes in platelet count and MPV or PDW levels

| Variable 1 | Variable 2 | Correlation coefficient | p value |
| --- | --- | --- | --- |
| Change in platelet count from baseline | Change in MPV from baseline |  |  |
| week 1 | week 1 | - | - |
| week 2 | week 2 | −0.50 | 0.21 |
| week 4 | week 4 | −0.05 | 0.91 |
| week 8 | week 8 | −0.85 | 0.007 |
| week 12 | week 12 | −0.71 | 0.047 |
| week 16 | week 16 | −0.31 | 0.54 |
| week 20 | week 20 | −0.68 | 0.09 |
| week 24 | week 24 | −0.61 | 0.15 |
| Change in platelet count from baseline | Change in PDW from baseline |  |  |
| week 1 | week 1 | - | - |
| week 2 | week 2 | −0.31 | 0.46 |
| week 4 | week 4 | −0.21 | 0.64 |
| week 8 | week 8 | −0.81 | 0.015 |
| week 12 | week 12 | −0.71 | 0.047 |
| week 16 | week 16 | −0.14 | 0.79 |
| week 20 | week 20 | −0.71 | 0.07 |
| week 24 | week 24 | −0.82 | 0.023 |

The correlation between the change in platelet count from baseline and the change in MPV or PDW from baseline was assessed using Spearman’s rank correlation coefficient.

MPV: mean platelet volume, PDW: platelet distribution width.

Supplementary Figure 1. Change in median platelet count after FOS initiation in patients whose platelet count at baseline was <30,000 / μL

A: All registered patients; B: Subgroup analysis with/without combination therapy with TPO-RAs; C: Subgroup analysis by treatment line with FOS.

Data are presented as median as line graph and interquartile range as area graph.

FOS: fostamatinib, TPO-RA: thrombopoietin receptor agonist

Supplementary Figure 2. Proportion of patients who achieved the platelet endpoint

A: Proportion of patients who achieved a platelet count ≥30,000 /μL and a twofold increase from baseline at least once after FOS initiation (cumulative).

B: Proportion of patients who achieved a platelet count ≥50,000 /μL at least once after FOS initiation (cumulative) (excluded patients whose platelet count was ≥50,000 /μL at baseline).

C: Proportion of patients who achieved a platelet count ≥100,000 /μL at least once after FOS initiation (cumulative).

FOS: fostamatinib

Supplementary Figure 3. Proportion of patients who met platelet endpoints at each observation point

A: Proportion of patients who achieved a platelet count ≥30,000 /μL and twofold increase from baseline at each observation point.

B: Proportion of patients who achieved a platelet count ≥50,000 /μL at each observation point (excluded patients whose platelet count was ≥50,000 /μL at baseline).

C: Proportion of patients who achieved a platelet count ≥100,000 /μL at each observation point.

FOS: fostamatinib
